# Variability of the nutrient stream near Kuroshio's origin

**DOI:** 10.1038/s41598-021-84420-5

**Published:** 2021-03-03

**Authors:** Chen-Tung Arthur Chen, Ting-Hsuan Huang, Chi-Hsuan Wu, Haiyan Yang, Xinyu Guo

**Affiliations:** 1 Department of Oceanography, National Sun Yat-Sen University, Kaohsiung, 804 Taiwan; 2 Ocean College, Zhejiang University, Zhoushan, 316021 Zhejiang China; 3 Taiwan Ocean Research Institute, National Applied Research Laboratories, Kaohsiung, 801 Taiwan; 4 Center for Marine Environmental Studies, Ehime University, 2-5 Bunkyo-Cho, Matsuyama, 790-8577 Japan

**Keywords:** Climate change, Ocean sciences

## Abstract

The Kuroshio—literally “the Black Stream”—is the most substantial current in the Pacific Ocean. It was called the Black Stream because this oligotrophic current is so nutrient-poor in its euphotic zone that the water appears black without the influence of phytoplankton and the associated, often colored dissolved organic matter. Yet, below the euphotic layer, nutrient concentrations increase with depth while current speed declines. Consequently, a core of maximum nutrient flux, the so-called nutrient stream, develops at a depth of roughly between 200 and 800 m. This poorly studied nutrient stream transports nutrients to and supports high productivity and fisheries on the East China Sea continental shelf; it also transports nutrients to and promotes increased productivity and fisheries in the Kuroshio Extension and the subarctic Pacific Ocean. Three modes of the Kuroshio nutrient stream are detected off SE Taiwan for the first time: one has a single-core; one has two cores that are apparently separated by the ridge at 120.6–122° E, and one has two cores that are separated by a southward flow above the ridge. More importantly, northward nutrient transports seem to have been increasing since 2015 as a result of a 30% increase in subsurface water transport, which began in 2013. Such a nutrient stream supports the Kuroshio's high productivity, such as on the East China Sea continental shelf and in the Kuroshio Extension SE of Japan.

## Introduction

**Figure 1:**
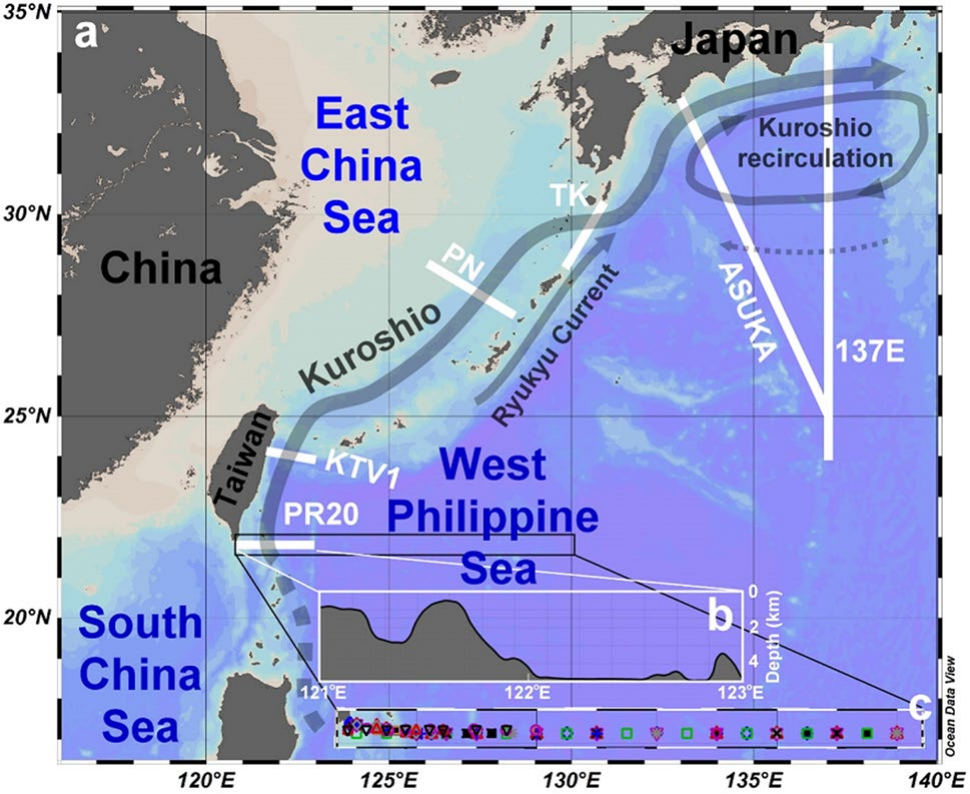
Study area. (**a**) The thick white lines represent different studied cross-sections, and the arrows denote the schematic current paths. (**b**) The bathymetry along the PR20 cross-section. (**c**) The sampled stations. The color symbols depict the sampling locations for cruises listed in Table S1.

The Kuroshio is an essential western boundary current and, since it is very nutrient-poor in its upper layer, biological productivity is low along its path. Yet, the Kuroshio subsurface waters are very nutrient-rich, and a nutrient stream reportedly exists below the euphotic zone^1–4^, similar to one that was first found in the Gulf Stream^5^. The spatial structure of such nutrient streams is unknown. Whether or how the enormous amounts of nutrients transported by the nutrient stream in the lightless zone contribute to ocean productivity warrants further study. This work concerns spatial and temporal variations of water and nutrient transports of the Kuroshio, and the reasons for such variations in the range 121–124° E at 21.75° N southeast of Taiwan, based on measurements made in 14 cruises between 1990 and 2015 (Fig. 1; Table S1). This cross-section was designated as the 20th Pacific Repeated Line in the World Ocean Circulation Experiment (PR 20).

### Nutrient stream and its structure modes

Supplementary Fig. 1 (Fig. S1) presents typical cross-sections of temperature (T), salinity (S), and concentrations of nitrate plus nitrite (N), phosphate (P), and silicate (Si). The temperature declines with increasing depth or σ_θ_ whereas the salinity reaches a maximum at about 200 m and a minimum at around 800 m. These extremes correspond to the tropical and intermediate waters, respectively. Concentrations of N, P, and Si all increase with depth and σ_θ_. All contours in the figure slant upward to the left, reflecting the general uplifting of subsurface waters towards the west.

Cross-sections of T, S, N, P, and Si exhibit slight temporal variations, except for the seasonal variation of T and S in the surface layer (not shown). The northward-flowing Kuroshio is concentrated between 121 and 122° E, and the currents are weaker between 122 and 123° E along PR 20. Between 123 and 124° E, the currents are feeble and random in both direction and speed, even with a small net southward flow. East of Taiwan, the Kuroshio is centered within one degree of the coast^6^. Northeast of Taiwan, the Kuroshio begins to exhibit considerable variability, even branching into two currents^7^ but the focus here is on the PR 20 line. Since the Kuroshio has a high velocity but low nutrient concentrations close to the surface, the top 250 m accounts for an average 60% of the water flow but only about 14% of the nutrient transports. Without exception, nutrient transports are highest at a depth of several hundred meters (σ_θ_ = 26–27 kg/m^3^) but, hitherto unknown, water and nutrient fluxes exhibit three distinctive modes.

**Figure 2:**
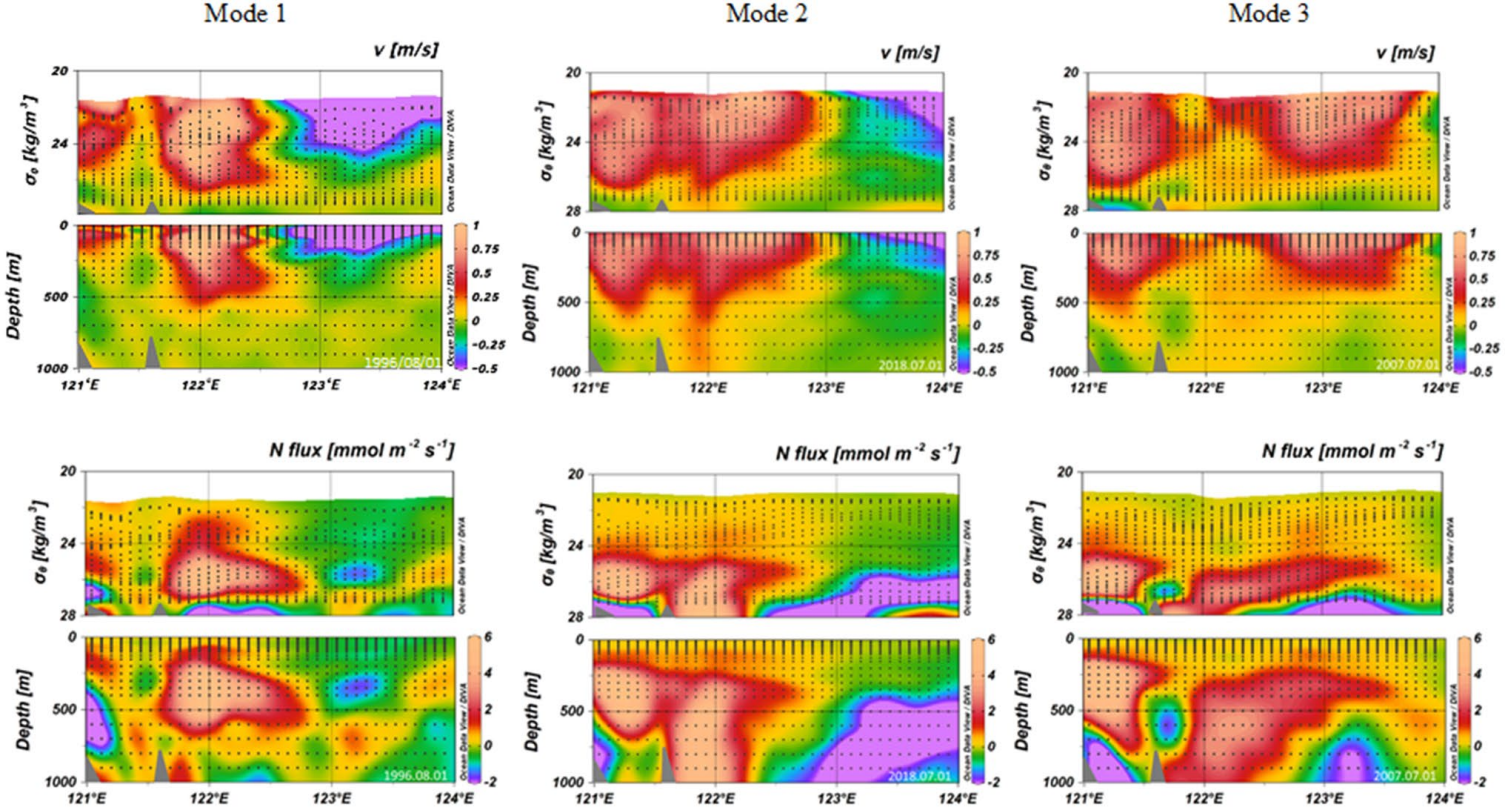
Typical northward velocity and N fluxes for Mode 1, 2, and 3 waters.

Figure 2 presents typical cross-sections of current velocity (v) and flux of N in these three modes. Figure S2 displays the P and Si fluxes. The nutrient flux structures were classified into three modes according to the core’s distribution for the maximum N flux. In line with velocity structures, there are single-core and two-core patterns. The two-core forms were further divided into two types based on whether a counter flow exists in the middle of two cores. Most of the flow between 121 and 123° E is northward, and the water flow is fastest above 500 m around 122° E. The nutrient streams are the most pronounced at about 122° E but are located at depths of 200–600 m for Mode 1 waters and 400–1000 m for Mode 2 and 3 waters. Mode 1 has a single dominating core, which is detected roughly 19% of the time. Concerning nutrient fluxes, Mode 1 transports 103 ± 88, 7.7 ± 6.6, and 149 ± 186 kmol/s of N, P, and Si, respectively, between 121 and 123° E. These fluxes are the lowest among the three modes. East of 123° E, the currents are weak and frequently flow southward.

The Kuroshio exhibits typically a single core along its path, but a two-core current structure has sometimes been detected^6,8,9^. Indeed, Mode 2 displays two connecting cores in terms of current spread and nutrient fluxes (Figs. 2 and S2). The western core of nitrate is present at water depths of 200–600 m but the eastern core close to 122° E extends to a depth of 1000 m or more. East of 123° E, weak southward currents, and nutrient fluxes are present. This mode is found most frequently, about 41% of the time, and transports 202 ± 97, 15 ± 7.3, and 348 ± 200 kmol/s of N, P, and Si, respectively, between 121 and 123° E. These fluxes are the highest among the three modes. The first report of the Kuroshio nutrient stream, by Chen et al.^1^, did not identify three modes but their Figs. 2 and 4 depict a two-core structure.

Mode 3 has a similar shape to Mode 2 but in between the two cores are southward flow and nutrient fluxes (Figs. 2 and S2). The multi-year average Acoustic Doppler Current Profiler data with 1/4° × 1/4° resolution (Ocean Data Bank of the Ministry of Science and Technology, Republic of China; http://www.odb.ntu.edu.tw/) confirms the weak southward flow of < 0.02 m/s between 200 and 300 m at about 121.6° E. The linkage of the separation of the two cores to the ridge at about 121.6–122° E, as suggested by Liang et al.^8^, is intuitive (Fig. 1b). Westward anticyclonic eddies and winter Kuroshio intrusion provide the other possible mechanisms to form the two-core structure^10,11^. Notably, the ridge's tip is at 121.6° E in the studied cross-section, but the southern part of the ridge centers at about 122° E. Mode 3 is detected approximately 32% of the time and transports 185 ± 118, 14 ± 8.8, and 324 ± 243 kmol/s of N, P, and Si, respectively, between 121 and 123° E. The three modes taken together are found 92% of the time, and at other times the flow is uncharacterized as the Kuroshio may have been more broken or there may have been transitions among various modes due to passing of eddies and other disturbances.

A subsurface front reportedly separates the water that flows out of the South China Sea (SCS) from the northward-flowing Kuroshio, which is composed of water from the West Philippine Sea (WPS)^12^. This front, extending from a water depth of 350 m to 1350 m, is centered at about 121.6–122° E, near the ridge. The θ/S characteristics of all waters west of 121.6° E are closer to those of the SCS water while those of waters east of 121.6° E are closer to those of the WPS water^12^. Accordingly, the core of Mode 1 and the eastern core of Modes 2 and 3 include mostly WPS waters, and the western cores of Modes 2 and 3 are primarily waters from the SCS.

### Variations of nutrient transports

The water and nutrient transports exhibit substantial variation but, on average, water transport is most significant in summer between 121 and 122° E (Fig. S3), perhaps reflecting the southward movement of the bifurcation point of the North Pacific Equatorial Current^13–15^. In spring and summer, 72% of the water transport above 250 m and 71% of the water transport between the surface and 1000 m is between 121 and 122° E in the region of 121–123° E. In fall and winter, the corresponding percentages are 66 and 64%, respectively. During the El Niño years, the higher summer transport between 121–122° E diminishes, but it increases during the La Niña years (Fig. S3). In El Niño years, between 122 and 123° E, water transport decreases substantially in summer. These results agree with the observation that in El Niño years, the bifurcation point moves northward, weakening the Kuroshio; the reverse is true in La Niña years^16,17^. Furthermore, in El Niño periods, the water transport between 121 and 122° E is high in summer but it is low between 122 and 123° E.

As mentioned above, nutrient transports above 250 m account for only around 14% of the total nutrient transport, and most of the transport is between 250 and 1000 m. Like water transport, most of the nutrient transport occurs between 121 and 122° E. The seasonality of the nutrient transports is similar to that of water transport and is not discussed here. The interannual variability is considered below.

**Figure 3:**
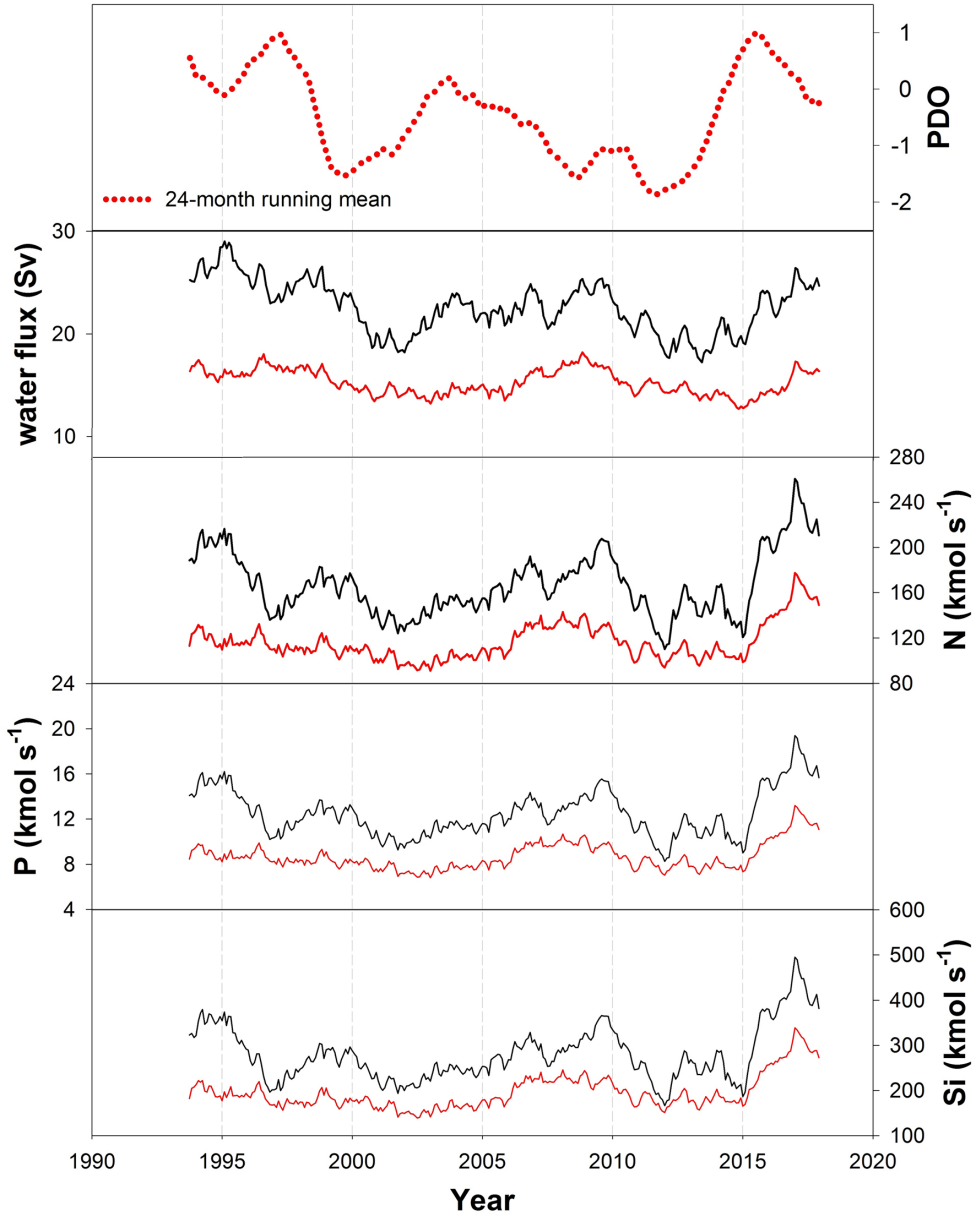
Interannual variation of the PDO index (24 month-moving-average), water, N, P, and Si transports (0–1000 m) between 121–123° E (black lines) and between 121–122° E (red lines).

Figure 3 presents the 24-month moving average of the water and nutrient transports across 121–122° E during the study period. The general patterns are decreasing from 1993 to 2013. However, the large increase, approximately doubling, of the nutrient transports after about 2015 is most notable. Water transport begins to increase around 2013, but the growth is only about 30%. The nutrient transports increased so much because, after 2013, the water transport below 250 m increased more than that above 250 m (Fig. S4a). As nutrient concentrations below 250 m are higher than those above, an increase in water transport below 250 m markedly increased the total nutrient transports (Fig. S4c).

The year 2013 marks the end of the global warming hiatus that began in 1998^18^. The Pacific Decadal Oscillation (PDO) index, which has been negative since 1998, also began to swing back to positive values in 2012/2013. Soeyanto et al.^19^ noted that the Kuroshio transport correlates positively with the PDO index from 1993 to 2002. This correlation disappears when the study period extends to 2012, perhaps because of intense mesoscale eddies after 2002 (Fig. 3). The low Kuroshio transport in 2013 was observed by the pressure-sensor-equipped inverted echo sounders and satellites^11,20^. The weakened Kuroshio related to the declined westerlies and cyclonic wind stress curl from 1993 to 2013^21^. The westerlies-driven Sverdrup transport increased from 2012 to 2017, so the enhanced Kuroshio is associated with the strengthening trade wind (Fig. S4b). The pattern of satellite sea surface height difference along 24.375° N is in line with the Sverdrup transport and HYCOM water transport results (Fig. S4). Unfortunately, we cannot yet fully explain why water transport increased after 2013 and was faster below 250 m than in the surface layer.

### Eventual consumption of nutrients

Nutrients transported by the Kuroshio nutrient stream do not contribute directly to primary production or fisheries because they are too deep in the water column to be utilized by phytoplankton. However, a small fraction of the Kuroshio Intermediate Water (the S_min_ layer) wells up to the East China Sea (ECS) continental shelf^22^, and becomes the primary source of nutrients on the ECS shelf^23^. Upwelled subsurface waters of the Kuroshio to the ECS shelf contain ten times the phosphate’s combined input from all rivers that enter the ECS^24^. Since the nutrient stream is located between the S_max_ and the S_min_ layers, part of it indeed contributes to the high productivity on the ECS shelf.

Chen^25^ established that the left part of the Kuroshio in the ECS comprises waters affected by the outflow from the SCS, which reaches about 122° E on the PR 20 line. As early as 1972, Nitani^26^ pointed out that the intermediate water from the SCS is around 100 m shallower than that in the WPS because of upwelling and intensive vertical mixing in the former. Once out of the SCS, the left part of the northward-flowing subsurface water upwells further to the left. In the ECS, another intense shoreward upwelling occurs, and the subsurface waters further upwell by 300 m within a degree in latitude (Fig. S5).

Based on the nutrient budgets of Chen and Wang^24^, only 4, 4 and 8% of the N, P, and Si, respectively, that are transported by the Kuroshio nutrient stream between 121 and 122° E are used on the ECS shelf, while the rest is transported farther to the north and northeast. Four reports on the Kuroshio nutrient stream along the Kuroshio path (Fig. 1) have been published. Close to the Kuroshio's origin is the PR 20 line (N, P, and Si; Chen et al.^1^ and this study), followed by the KTV1 line east of Taiwan^4^ (N only). Further downstream is the PN line in the East China Sea^2,3^ (N and P), and finally at three cross-sections south of Japan^2^ (TK, ASUKA, and 137° E lines; N only).

**Figure 4:**
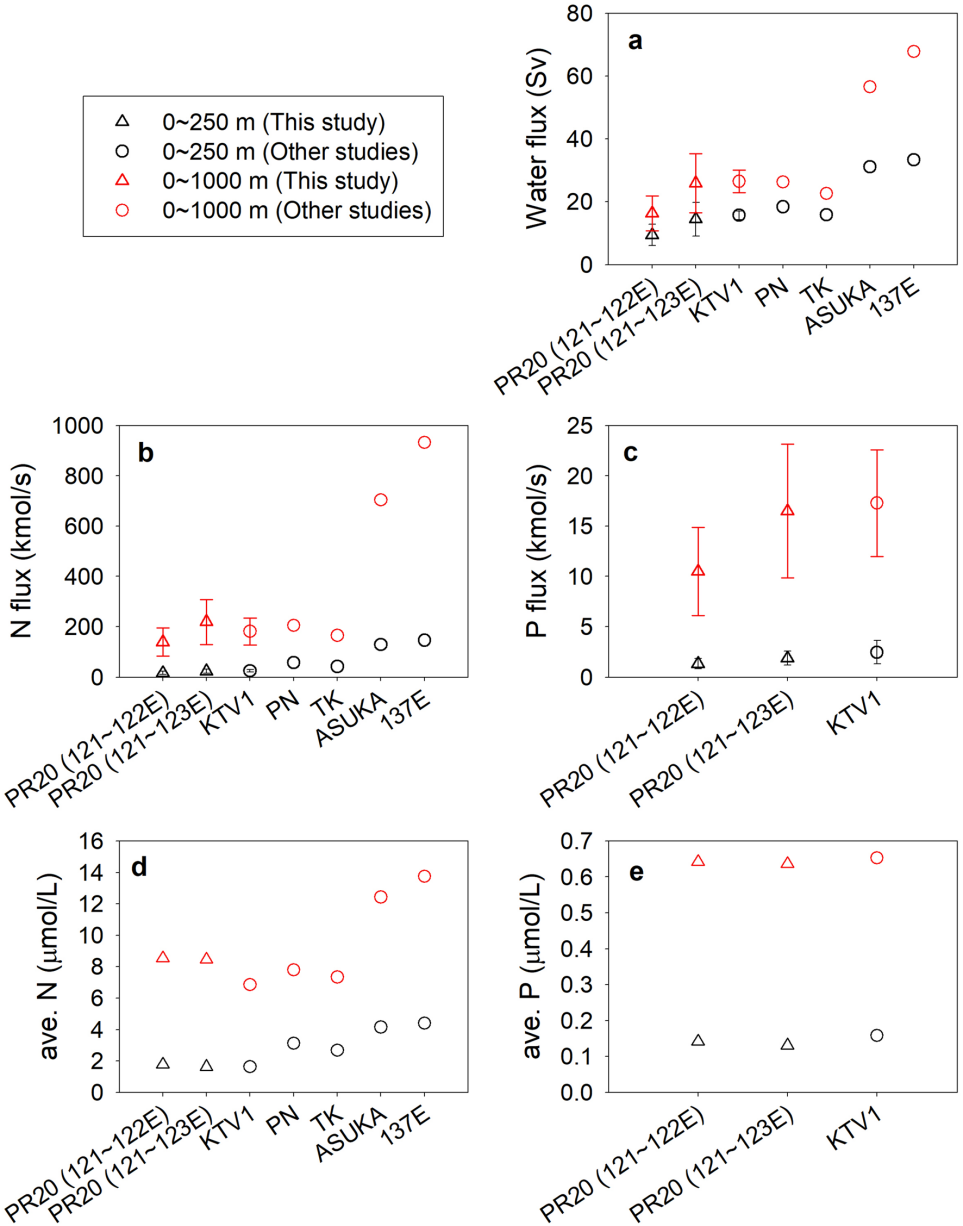
The poleward transports of (**a**) water, (**b**) N and (**c**) P and averaged (**d**) N and (**e**) P concentrations along the Kuroshio path. The data of KTV1 were calculated between 0–250 m and 0–1000 m^4^. These transports of PN, TK, ASUKA, and 137E were estimated^2,3^ for the layers between the surface and σ_θ_ = 25.5(280–350 m) or 27.3(850–1100 m).

The water transport at the PR 20 line (121–123° E) herein agrees with all other relevant studies within uncertainties (Fig. 4a) but is more than 50% less than that in the Gulf Stream^5,27^ (25 ~ 27° N). The nutrient transports herein also agree with reported values for the Kuroshio region, except for the very high N transport across the ASUKA line^2^. Since the nutrient concentrations beneath the Kuroshio and the Gulf Stream are similar, higher transports are associated with the latter due to its much higher velocity and more significant water transport. For the Kuroshio, the cross-section's water and nutrient transports agree to within 10% between PR 20 and PN lines. A lack of data makes other comparisons impossible.

An increase in the poleward N transport along the Kuroshio flow path is of particular interest (Fig. 4b), and significant enhancement exists between TK and ASUKA. The major mechanism is the merger with the northeastward Ryukyu Current and the minor contribution is from the Kuroshio recirculation (Fig. 1)^28,29^. There is an increase in poleward water transport especially between 250 and 1000 m (Fig. 4a) and this is associated with deepened Kuroshio from upstream to downstream^30,31^. The nutrient concentrations are generally higher with increasing depth so denser waters contain higher nutrient concentrations. As a result, the N-transport increased between TK and ASUKA mainly due to more high-N dense water merging into the Kuroshio. Consequently, the section-averaged (0–1000 m) N concentration jumps from 8.6 mmol m^−3^ at PR20 and 7.3 mmol m^−3^ at TK, to 12.4 mmol m^−3^ at ASUKA, and finally to 13.7 mmol m^−3^ at 137E (Fig. 4d). Poleward P transport trends and averaged P concentration are similar to the N results from PR20 to KTV1 (Fig. 4c, e).

Of note is that recently Uchiyama et al.^32^ reported that the Kuroshio Extension area has a 23.5% higher productivity than the Kuroshio region. Oka^33^ defined the Subtropical Mode Water formed in the Kuroshio Extension region as that between 16 and 19.5 °C (Fig. S6) with σ_θ_ in the range of 24.5–25.8 kg/m^3^. These ranges exist in the upper layers of the nutrient streams so vertical mixing is naturally concluded to have brought nutrients to the euphotic zone when the Kuroshio reaches and goes beyond the Subtropical Mode Water formation regions. Figure S7 displays the 2018 annual average chlorophyll a concentration in the NW North Pacific Ocean. The high values in the Mode Water formation region and a little south provide evidence of the supply of nutrients to the euphotic layer by processes such as standing cyclonic eddies between the southern Japan coastline and the Kuroshio. The Oyashio also plays a role especially in the northern part of the Kuroshio Extension area^32,34,35^. It is natural to suspect that the Mode water would upwell the most readily as the high nutrient core is the shallowest. Yet, whether or how the three modes of the Kuroshio nutrient stream affect the subsequent upwelling and primary production along the path of the Kuroshio warrants further study.

## Conclusions

Since the nutrient concentrations are low in the surface layer of the Kuroshio, in which the water transport is high, and the nutrient concentrations are elevated in deep layers where the water transport is low, a "nutrient stream" develops at a depth of several hundred meters. At the PR 20 line SE of Taiwan, the nutrient transports between 121 and 122° E exceed those between 122 and 123° E. This nutrient stream has three modes—Mode 1 with a single-core, Mode two with two cores separated at 121.6° E, and Mode 3 with two cores and a return flow at 121.6° E. Mode 2 occurs the most frequently and has the highest fluxes.

Between 121 and 122° E, fluxes exhibit large seasonal variations; the fluxes are highest in summer when the bifurcation of the North Pacific Equatorial Current shifts southward. The 24-month moving average reveals that from 2015, the nutrient fluxes have increased significantly but the total water transport has only increased slightly since 2013, because although the speed of the current in the top layer above 250 m has fallen, that between 250 and 1000 m has increased. The nutrient stream has also been detected along the Kuroshio flow path downstream of the study area herein, SE of Taiwan^3^. A small part of the upper nutrient stream upwells to the ECS shelf and supports high productivity there. Some remaining nutrients are transported and vertically mixed up to the euphotic layer south and southeast of Japan, contributing to the high productivity in the Kuroshio Extension region^36^. Enhanced nutrient transport, reported herein, may become important shortly, affecting biological productivity and fish catches.

## Methods

### Physical data

Nutrient transports were estimated using the modeled water transport and simulated chemical concentrations. The daily salinity (S), temperature (T), and flow velocity were obtained using the Hybrid Coordinate Ocean Model (HYCOM) of the U.S. Naval Oceanography Office (https://www.hycom.org/data/glbu0pt08). The wind force in the HYCOM system is obtained from the Climate Forecast System Reanalysis. The analysis herein is from Oct. 1992 to Nov. 2018 and the transports calculated are at 21.76° N from 121 to 124° E. The horizontal resolution is 0.08° and the depths are 0, 2, 4, 6, 8, 10, 12, 15, 20, 25, 30, 35, 40, 45, 50, 60, 70, 80, 90, 100, 125, 150, 200, 250, 300, 350, 400, 500, 600, 700, 800, 900 and 1000 m. Data from the first day of each month were used.

To evaluate the S and T values in the model, in situ S and T data were compared with the model’s values at the same depth and similar locations. The in situ S and T data were collected in the region 121–130° E, 21.7–22° N from Oct. 1990 to Aug. 2015. A total of 14 cruises were carried out on R/V Ocean Research vessels I, II, and III (Table S1). Supplementary Fig. S8 displays the differences between the HYCOM results and the measured values. The mean differences (HYCOM result – measured value) in S and T are 0.013 ± 0.163 and 0.127 ± 1.143 °C, respectively. Generally, the HYCOM salinity values are higher than measured values, and the S differences decrease with increasing depth from 0 to 150 m. Most of the HYCOM temperature values are higher than the CTD values.

### Chemical data

A total of 372 bottle samples were obtained using a CTD/Rosette sampler. The concentrations of N, P, and Si were determined using published methods. The N concentration was determined using the pink azo dye method, with a precision of approximately ± 1 and ± 3% at 35 and 1 μmol kg^−1^, respectively. The P concentration was determined using the molybdenum blue method with a precision of approximately ± 0.5 and ± 3% at 2.5 and 0.1 μmol kg^−1^, respectively. The Si concentration was obtained using the silicon molybdenum blue method with a precision of around ± 0.6 and ± 2% at 150 and 5 μmol kg^−1^, respectively.

### Regression formula

To estimate concentrations of chemicals, linear, quadratic, and cubic regression equations were derived from in situ T and measured chemical concentrations. Using different water masses in the study area, empirical formulas for chemical concentrations were acquired using measured physical and chemical data at various T. Two sets of formulas were obtained for waters with the temperature above and below 20 °C, respectively. Supplementary Table S2 presents the coefficients of regression fits, adjusted coefficients of determination, and residual standard errors.

### Contribution of predictor variables

The estimated chemical parameter concentration was calculated using the mentioned regression formulas and modeled daily temperature of HYCOM. To evaluate the contribution of T to the empirically determined chemical concentrations, correlations between simulated chemical values and different prediction parameters were compared. Supplementary Fig. S8 displays the differences in the measured values and the estimated data for in situ temperature and HYCOM modeled temperature. As mentioned in the “Physical Data” section, the HYCOM temperature values slightly exceed the measured values. The overestimation of T is responsible for the underestimation of nutrient concentrations. Yet, supplementary Fig. S8 shows little difference between the average measured and estimated data.

### Assumptions made in the regression formula

The following assumptions are made. (1) Biological production and consumption do not vary within a season for fixed T, and (2) any effect of air temperature variations is negligible.

## Supplementary Information

Supplementary Information

## Data Availability

All data generated during this study are available in the ocean data bank (www.odb.ntu.edu.tw).
