# Supplementary material for: Variability of the nutrient stream near Kuroshio's origin
Source: Sci Rep. 2021 Mar 3;11:5080. doi: 10.1038/s41598-021-84420-5 (PMC7930021; doi:10.1038/s41598-021-84420-5)
Supplement: Supplementary file 1 — Supplementary Information [file 41598_2021_84420_MOESM1_ESM.docx]

**Supplementary Materials for**

**Variability of the nutrient stream near Kuroshio's origin**

Chen-Tung Arthur Chen, Ting-Hsuan Huang, Chi-Hsuan Wu, Haiyan Yang, Xinyu Guo

Correspondence to: ctchen@mail.nsysu.edu.tw

tinghhuang@narlabs.org.tw

Table S1 List of Cruises.

|  | Winter | Spring | | Summer | | | Autumn | |
| --- | --- | --- | --- | --- | --- | --- | --- | --- |
| Month/  Year | Jan. | Mar. | May | Jun. | Jul. | Aug. | Sept. | Oct. |
| 1990 |  |  |  |  |  |  |  | ORI-257 |
| 1991 |  |  |  | ORI-287 |  |  |  |  |
| 1992 |  |  | ORI-316 |  |  |  |  |  |
| 1994 |  |  | ORI-387 |  |  |  |  |  |
| 1995 |  |  | ORI-418 |  |  |  |  | ORI-433 |
| 1996 |  |  |  |  |  |  | ORI-462 |  |
| 2004 |  |  |  |  | ORI-725 |  |  |  |
| 2006 | ORⅢ-1126 |  | ORⅢ-1149 |  |  |  |  |  |
| 2007 |  |  |  |  | ORI-837 |  |  |  |
| 2009 |  | ORⅢ-1347 |  |  |  |  |  |  |
| 2011 |  |  |  | ORⅢ-1546 |  |  |  |  |
| 2015 |  |  |  |  |  | ORⅢ-1875 |  |  |

Table S2 Simulated chemical equation and corresponding mean difference between fitted data and measured data

| Parameter  (μmol/L) | Temperature range (°C) | Equation | mean difference between fitted data (in situ temp.) and measured data |
| --- | --- | --- | --- |
| N | 4~20 | y= 0.0606x^2^ – 3.7599x + 52.936 | -0.06±1.62 |
|  | ≥20 | y= -0.1828x + 5.634 | 0.32±0.55 |
| P | 4~20 | y= 0.0048x^2^ – 0.2862x + 3.9631 | 0.003±0.12 |
|  | ≥20 | y= -0.0139x + 0.4373 | -0.0001±0.05 |
| Si | 4~20 | y= -0.0288x^3^ + 1.6153x^2^ – 31.739x + 221.49 | -0.006±6.78 |
|  | ≥20 | y= -0.143x + 5.29 | -0.15±0.83 |

(d)

(e)

(c)

(a)


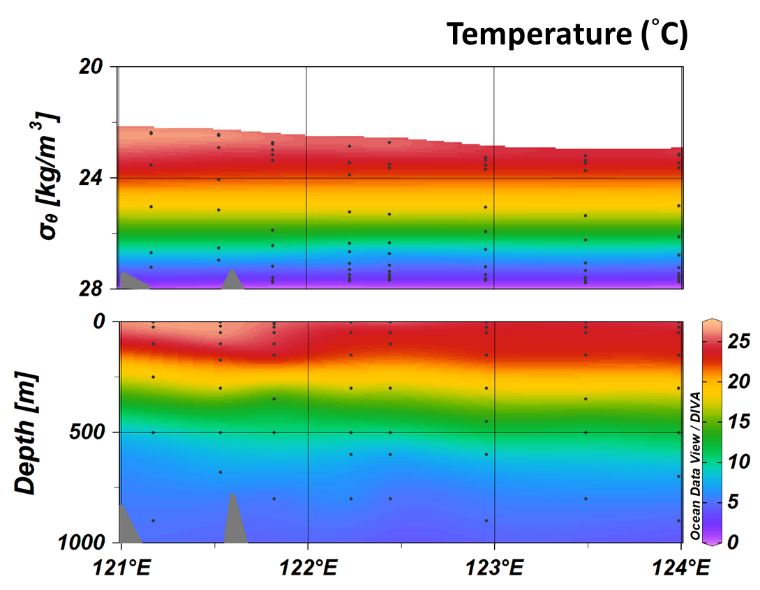

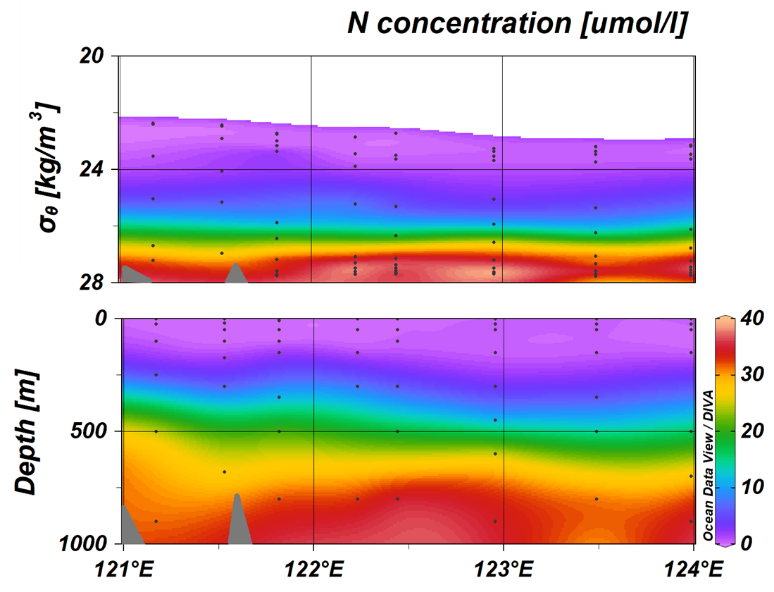

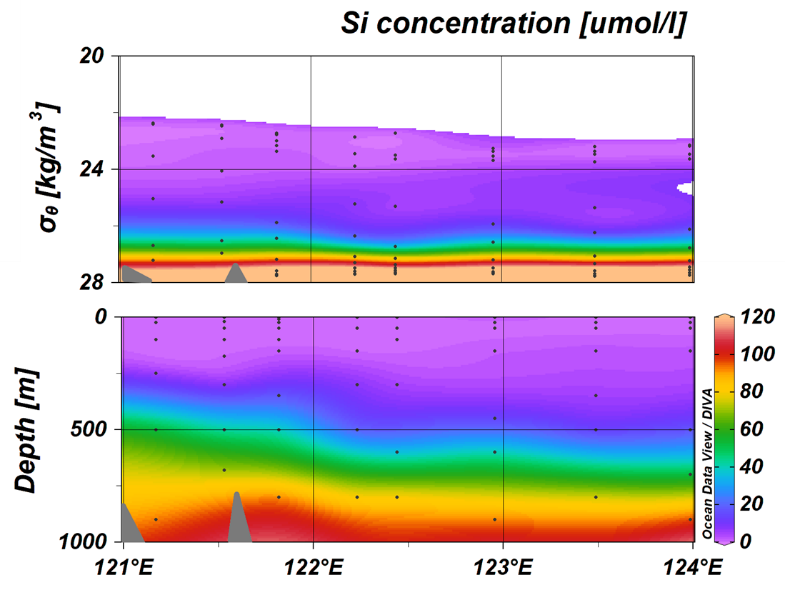


(b)


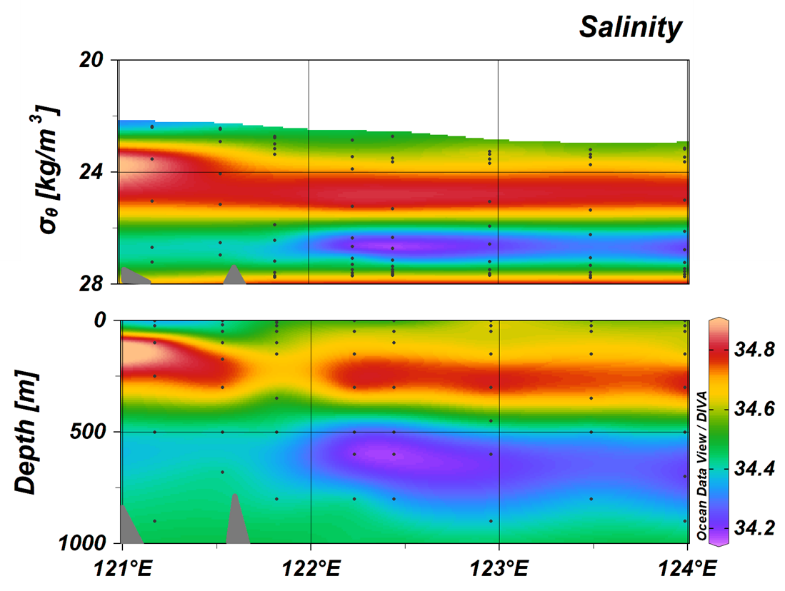

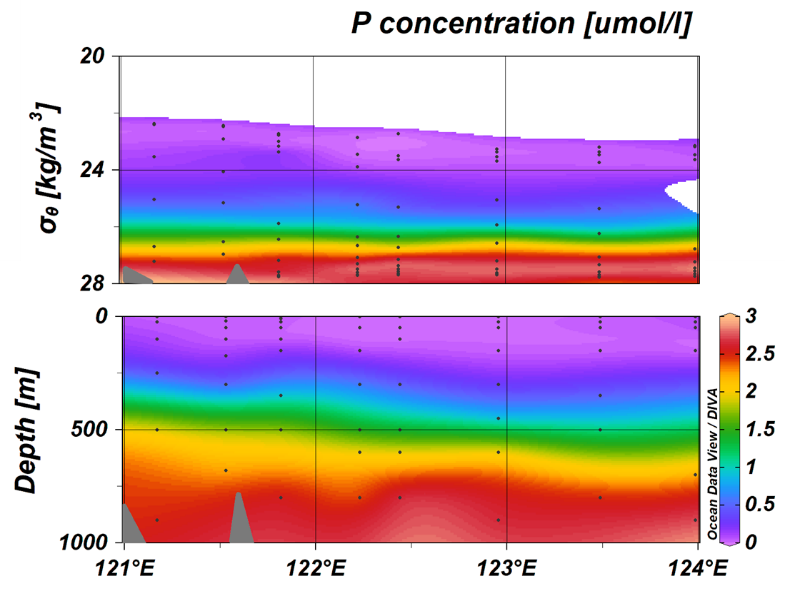


Figure S1 Typical a) temperature, b) salinity, c) N, d) P and e) Si cross-sections (data from March 2009, ORIII-1347 cruise).

Mode 1 Mode 2 Mode 3


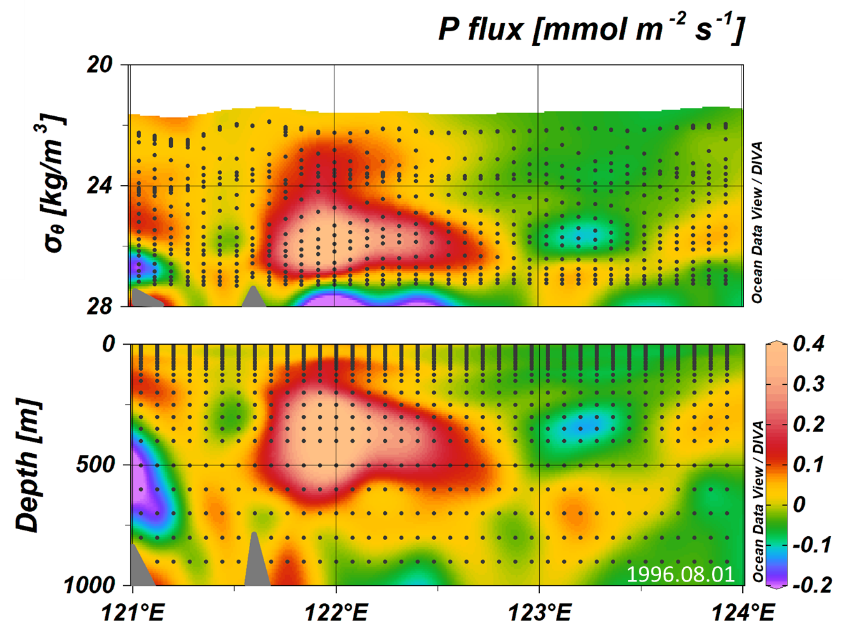

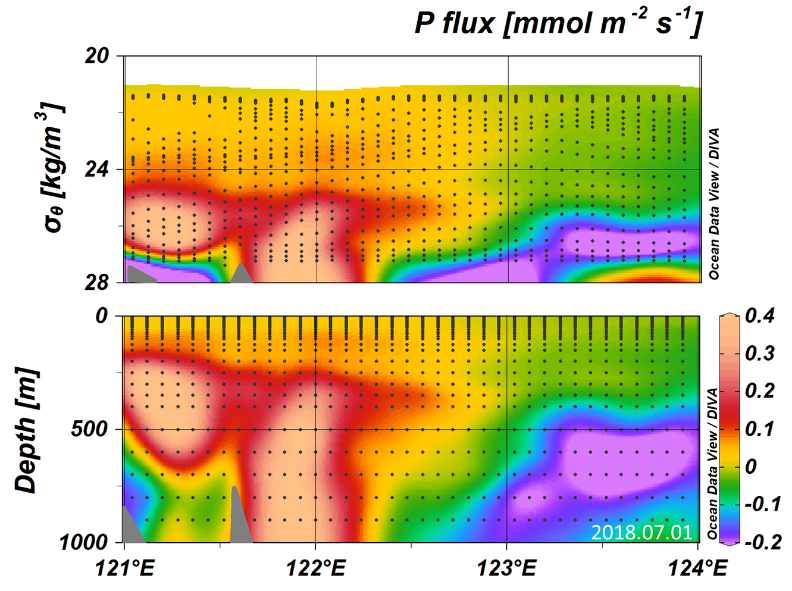

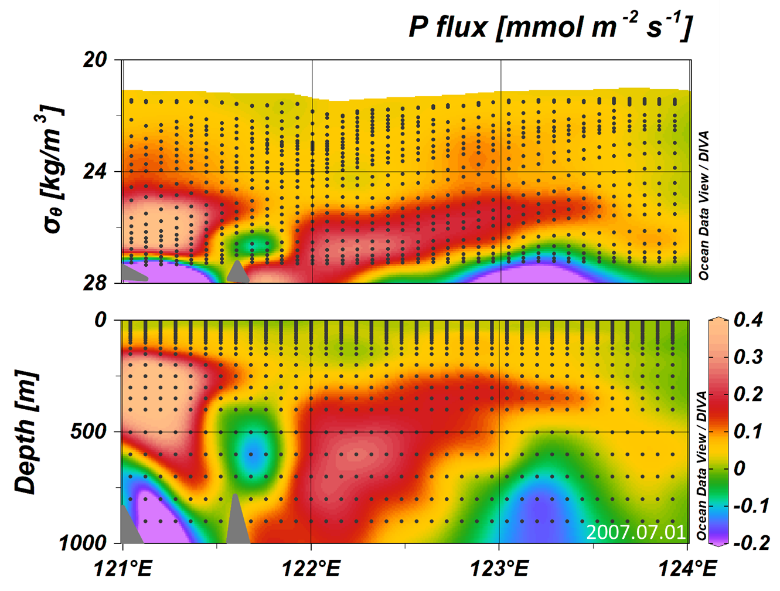

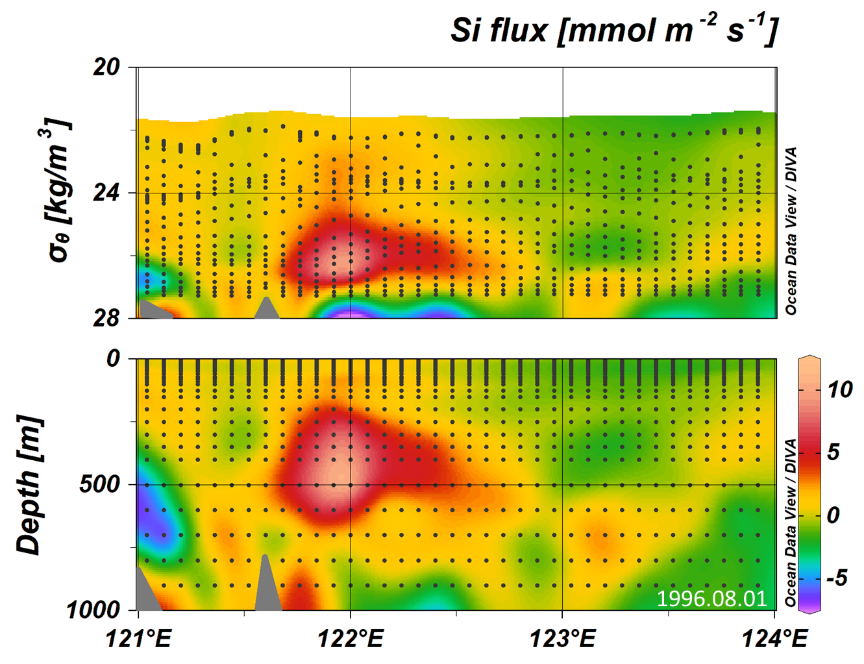

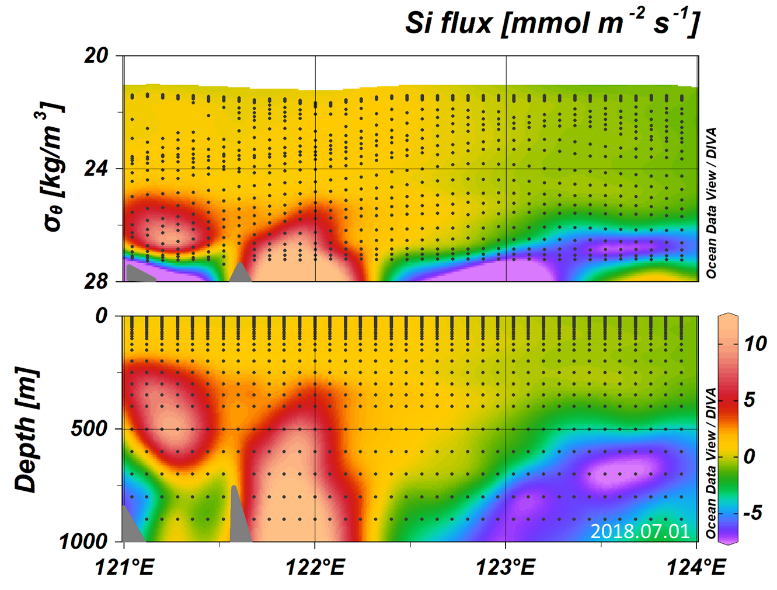

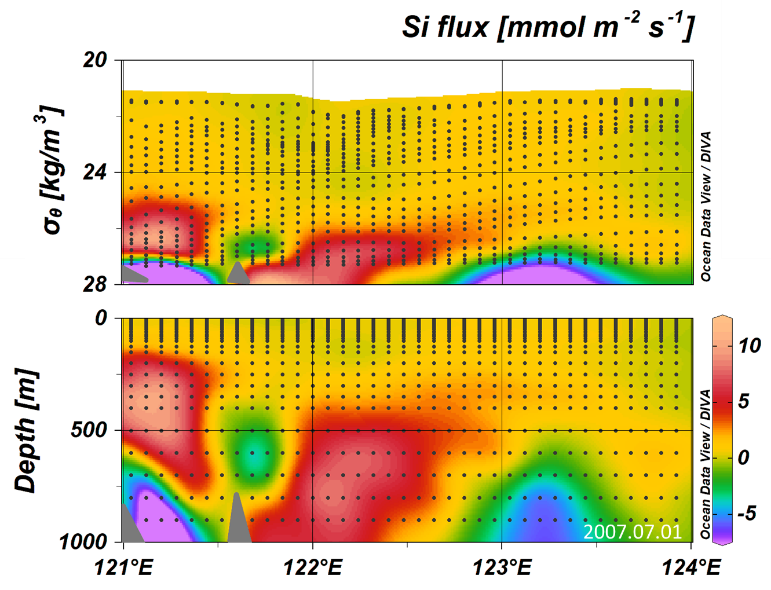


Figure S2 Typical P and Si fluxes for Mode 1, 2 and 3 waters.


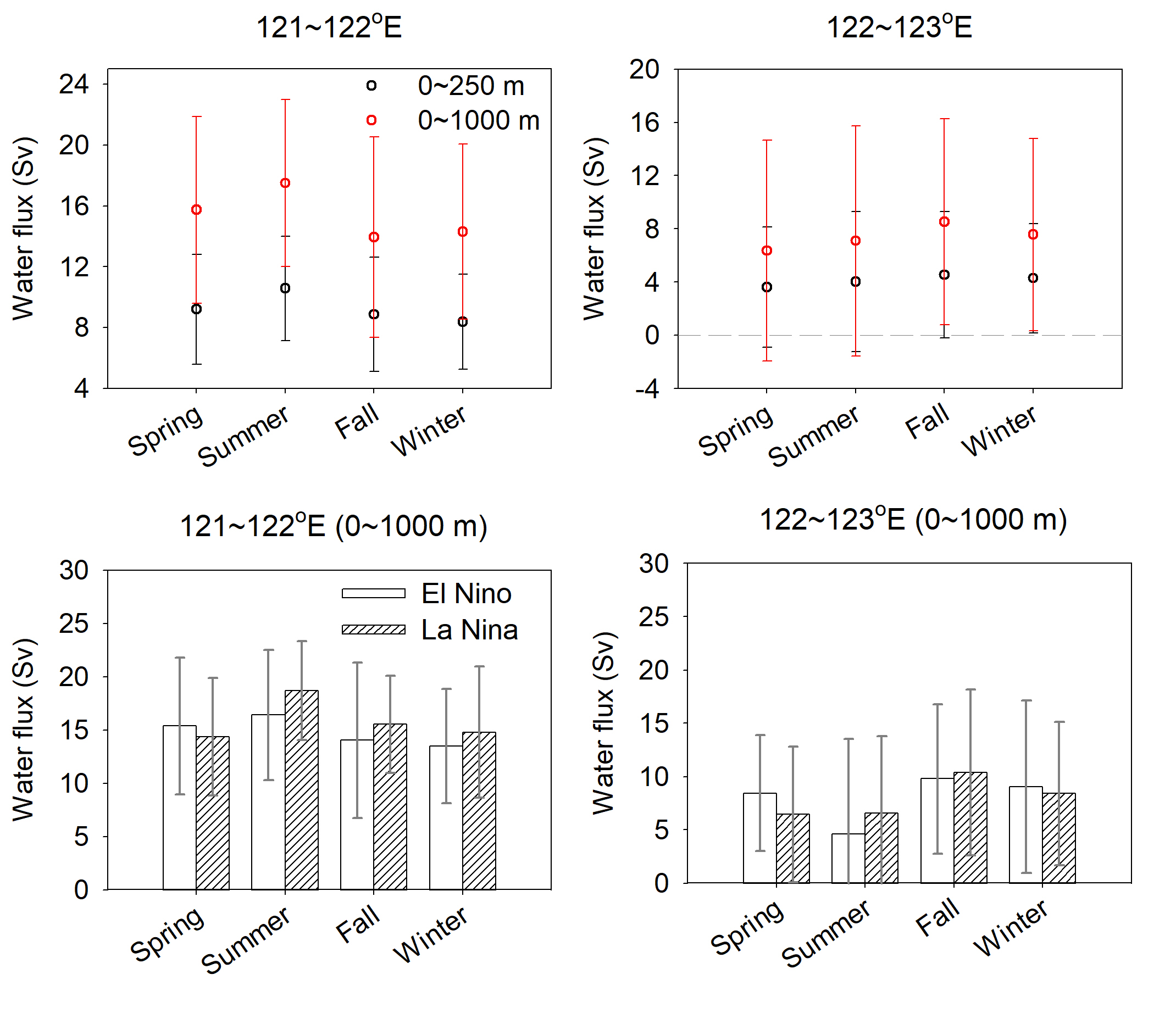


Figure S3 Seasonal fluxes of water in various longitude bands and different climate events.


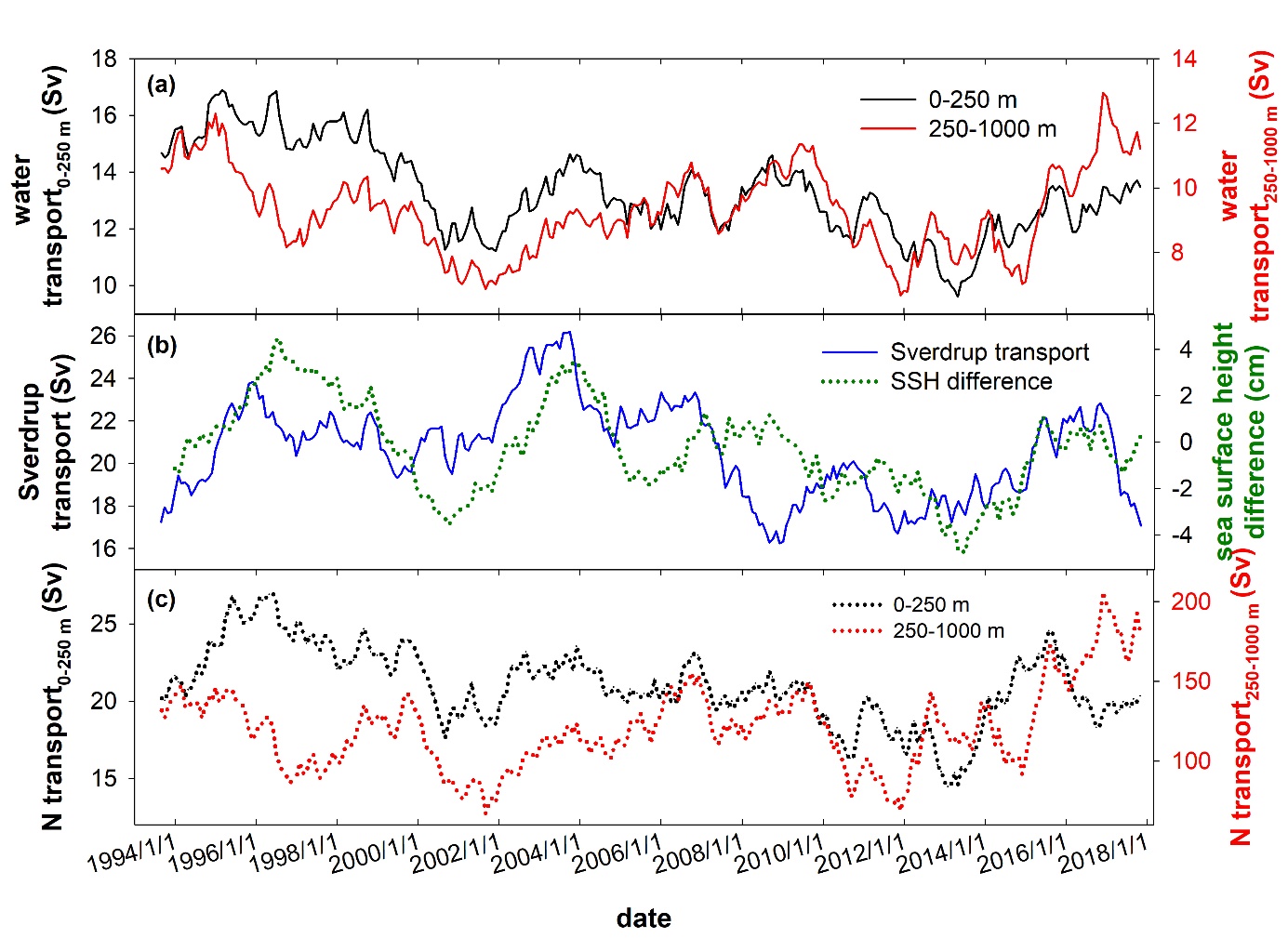


Figure S4 Twenty-four month-moving-averages of a) water transport, b) Sverdrup transport driven from NCEP 6-hourly wind and satellite sea surface height difference between Iriomote Island (123.625°E, 24.375°N) and Suao (121.875°E, 24.375°N), and c) N transports across 121 -124°E in the 0-250 m (black) and 250-1000 m (red) layers.


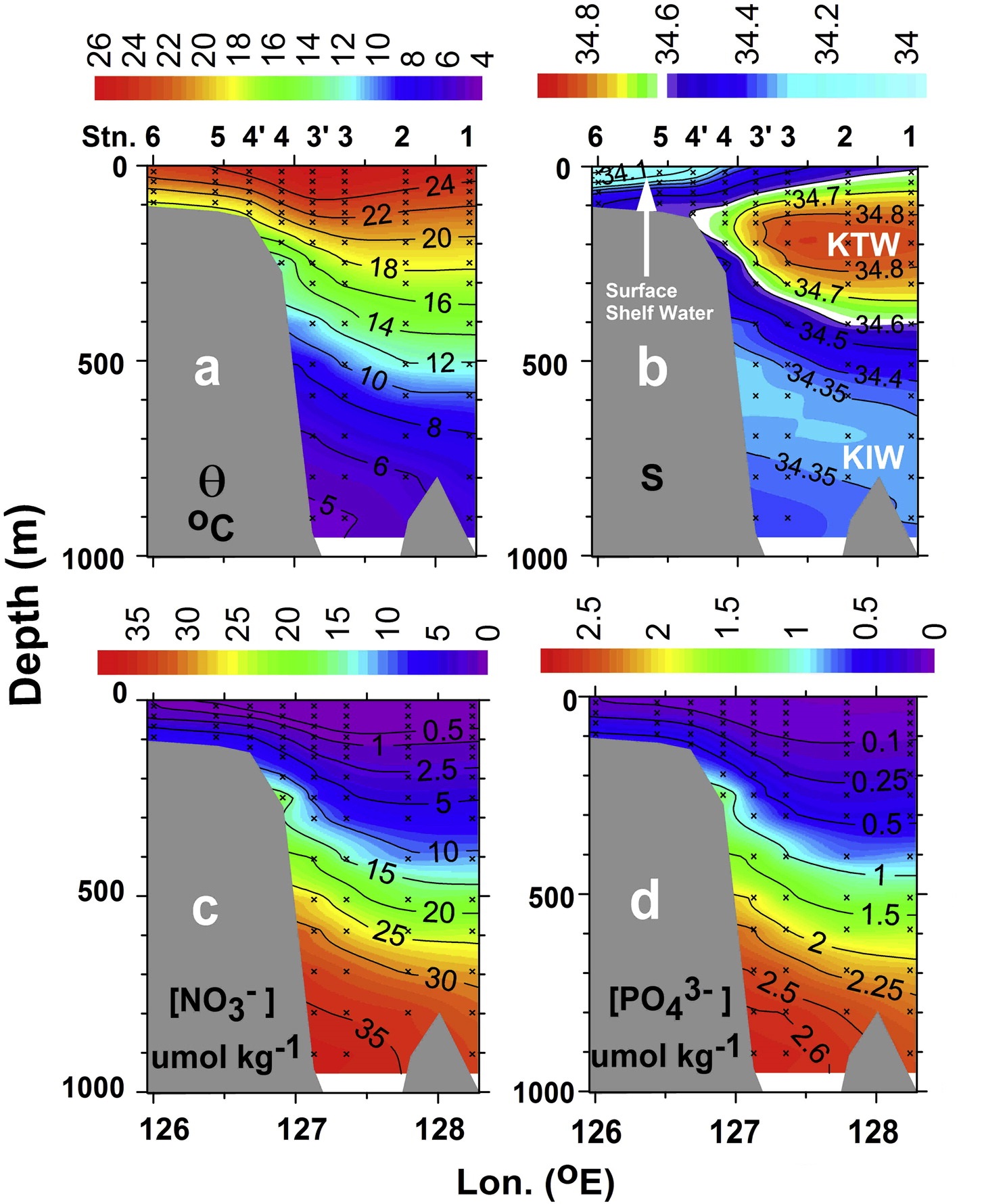


Figure S5 Distributions of typical (a) θ, (b) S, (c) NO_3_^−^, and (d) PO_4_^3−^ concentrations at the PN line (taken from Lui et al., 2014).


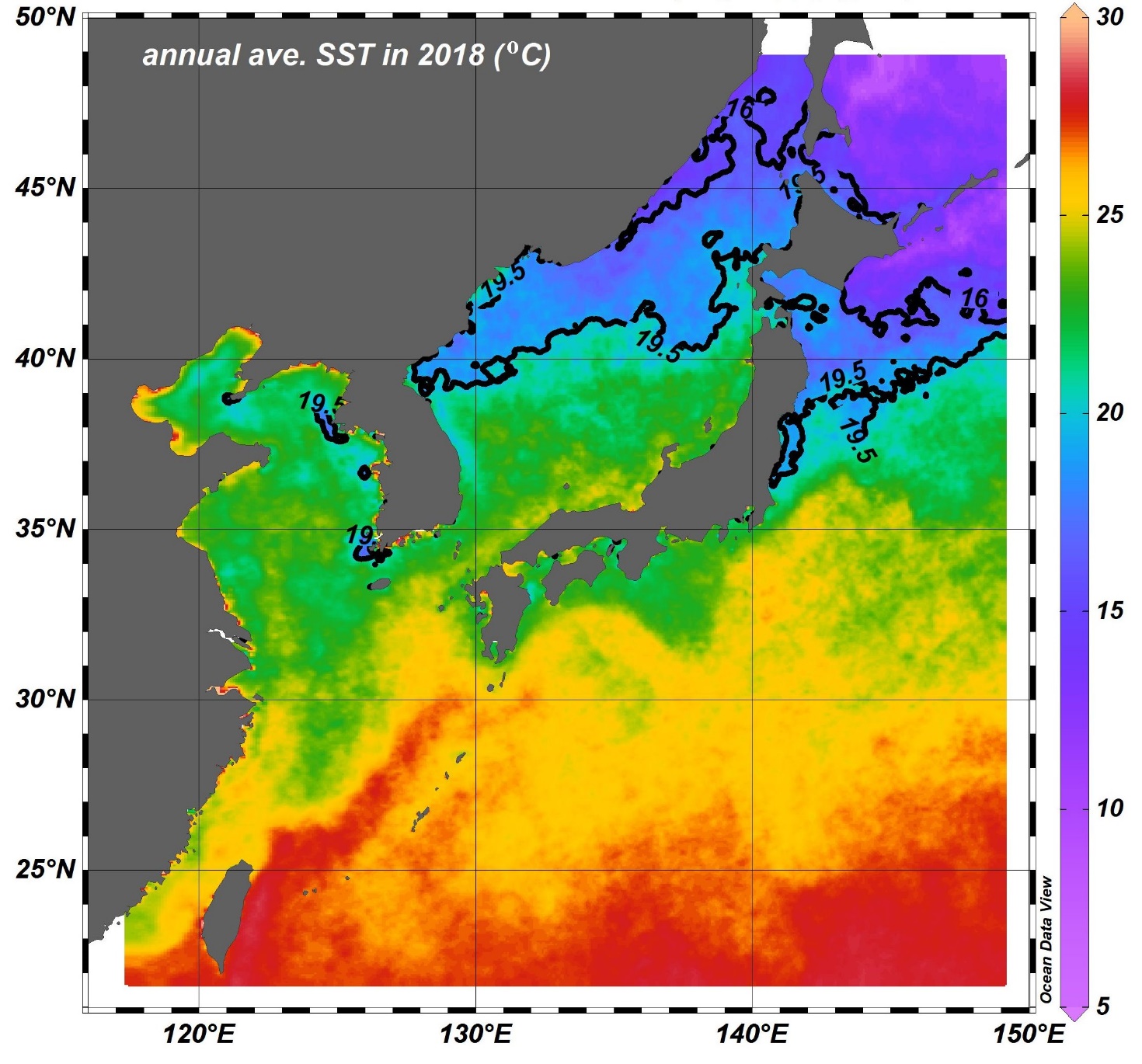


Figure S6 The region with an average annual sea surface temperature between 16 and 19.5°C in the North Pacific Ocean during 2018 (the data was adopted from https://worldview.earthdata.nasa.gov/, and the figure was generated from ODV, version 5.3.0, Schlitzer, Reiner, Ocean Data View, https://odv.awi.de, 2020).


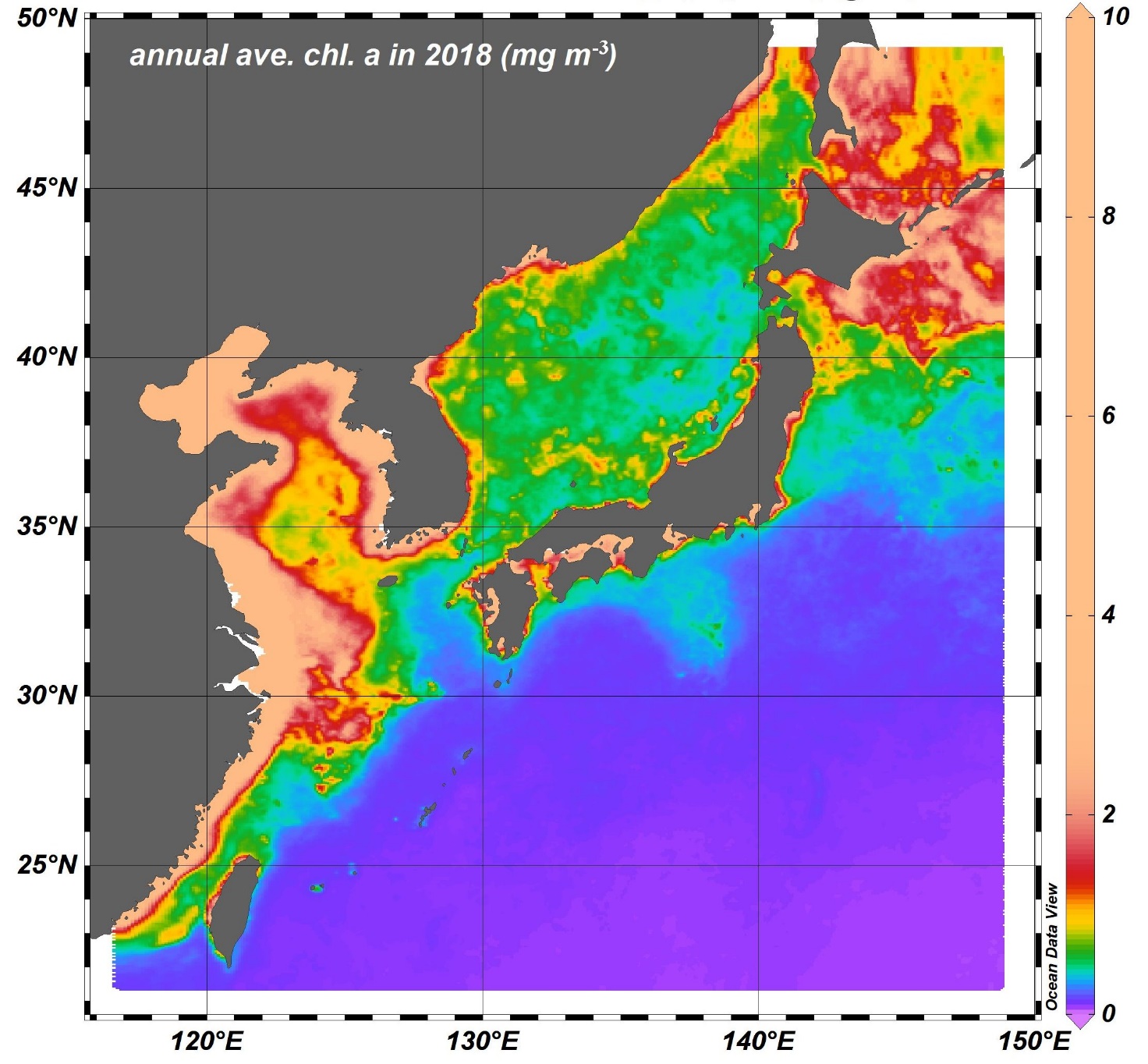


Figure S7 The annual average chlorophyll a concentration in the North Pacific Ocean during 2018 (the data were adopted from https://oceancolor.gsfc.nasa.gov/l3/, and the figure was generated from ODV, version 5.3.0, Schlitzer, Reiner, Ocean Data View, https://odv.awi.de, 2020).


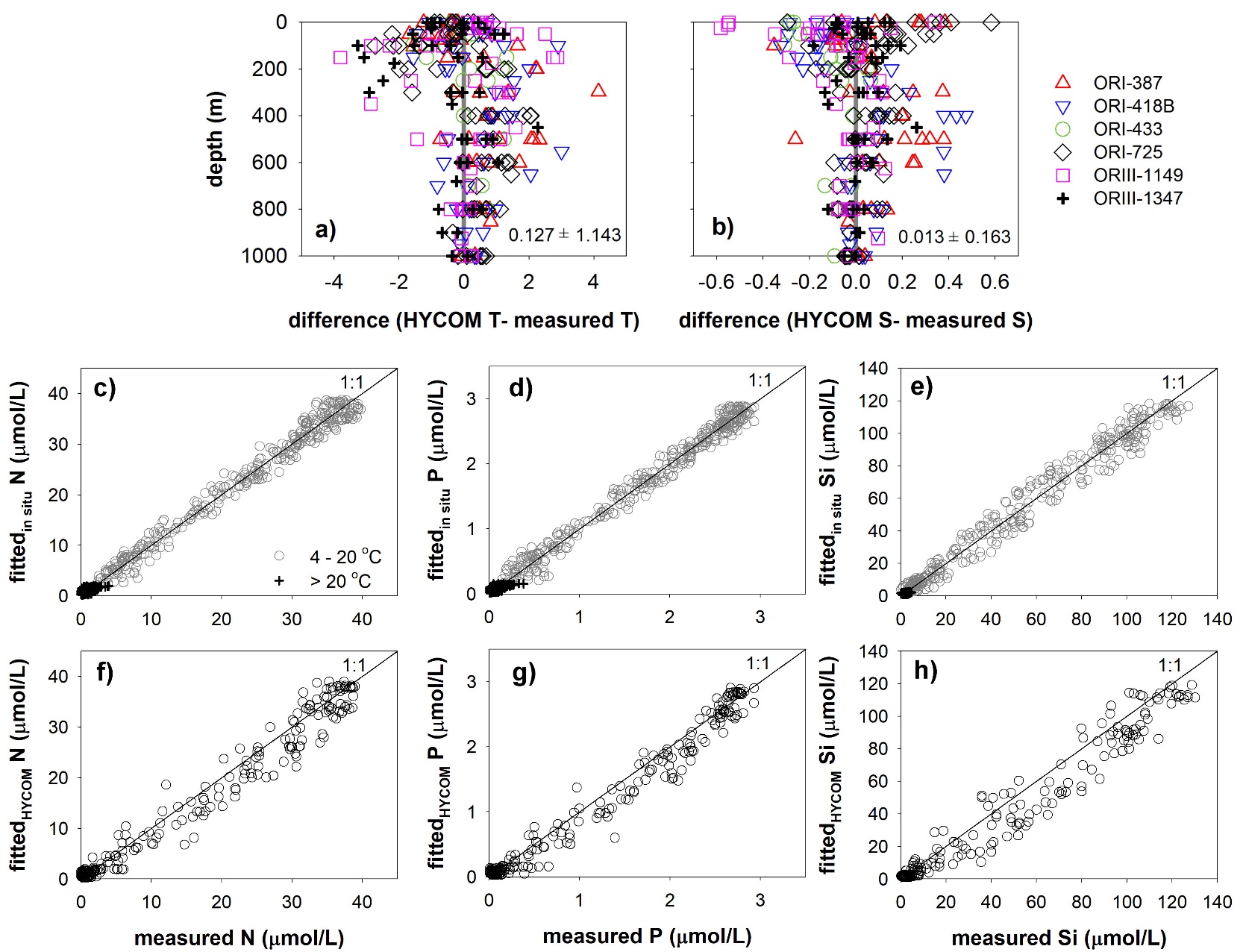


Figure S8 Difference between the in situ data and a) HYCOM model temp. and b) model S. The correlations between measured nutrient concentrations and fitted results based on c-e) in situ temperature and f-h) HYCOM model temperature.
